# Supplementary material for: Heterogeneous peer effects of college roommates on academic performance
Source: Nat Commun. 2024 Jun 6;15:4785. doi: 10.1038/s41467-024-49228-7 (PMC11156860; doi:10.1038/s41467-024-49228-7)
Supplement: Supplementary file 1 — Supplementary Information [file 41467_2024_49228_MOESM1_ESM.pdf]

Supplementary Information:

Heterogeneous peer effects of college roommates on academic performance

Yi Cao[1,2], Tao Zhou[1,2,*], Jian Gao[3,4,5,6,*]

[1] CompleX Lab, University of Electronic Science and Technology of China, Chengdu, China
[2] Big Data Research Center, University of Electronic Science and Technology of China, Chengdu, China
[3] Center for Science of Science and Innovation, Northwestern University, Evanston, IL, USA
[4] Kellogg School of Management, Northwestern University, Evanston, IL, USA
[5] Northwestern Institute on Complex Systems, Northwestern University, Evanston, IL, USA
[6] Faculty of Social Sciences, The University of Hong Kong, Hong Kong SAR, China
[*] Correspondence to zhutou@ustc.edu or jian.gao1@kellogg.northwestern.edu

Table of Contents

1. Data on student roommate and academic performance 2
   1.1 Dorm rooms in Western and Chinese universities 2
   1.2 Roommate assignment in university dorms 3
   1.3 Student accommodation and academic performance data 4
2. Tier combinations of roommate academic performance 5
   2.1 The theoretical probability for each combination 5
   2.2 Comparison between actual and theoretical probabilities 6
3. Roommate null model and statistical test 7
   3.1 Construction of a roommate null model 7
   3.2 Statistical hypothesis testing 8
4. Assimilation of roommate academic performance 9
   4.1 Academic performance assimilation 9
   4.2 Statistical tests for differences in assimilation 9
   4.3 Robustness check for the assimilation results 10
   4.4 The assimilation results by different gender 10
5. Regression models and regression results 10
   5.1 Regression on the effects of peer heterogeneity 10
   5.2 Regression on the effects of in-dorm ordinal rank 12
   5.3 Falsification tests of the regression results 13
Supplementary References 14
Supplementary Figures 17
Supplementary Tables 26

1. Data on student roommate and academic performance

We collect the accommodation and academic performance data of undergraduates from a public research-intensive university in China. In the university, students live in identical 4-person dorm rooms during their entire undergraduate program after a one-time plausible random assignment of roommates upon registration before the first semester. This randomization allows us to study the impact of college roommate peers on academic performance. In the following, we first introduce the differences in the layouts of a typical student dorm room in Western universities and in Chinese universities. We then introduce the roommate assignment process in the Chinese university and the longitudinal datasets of student accommodation and academic performance.

1.1 Dorm rooms in Western and Chinese universities

An on-campus dormitory, or residence hall, usually contains a large number of dorm rooms, which provide a place for students to sleep, offering opportunities for their educational and personal development[1-7]. Different from open and dynamic education environments such as classrooms[8-11], dorm rooms provide a close-knit living environment for students to interact and potentially learn from each other[12-14]. Arguably, while dorm rooms are not the primary places in which learning takes place such as classrooms and university libraries, they offer a highly interpersonal and spillover environment for a small group of student peers. This non-learning-oriented dormitory environment is ideally suited for studying the impact of student peers on learning outcomes.

In most Western countries, first-year undergraduates are required to live in on-campus dormitories for a certain period of time[14-18]. After this period, students have the option to continue living on-campus for the next academic year or to live off-campus. In the Western university dormitory, a typical dorm suite contains several bedrooms, where each student occupies a single bedroom (where a room contains only one bed) and shares public areas with other suite mates including living room, kitchen, and bathroom. In Supplementary Figure 1a, we show the floor plan of a typical Western university dorm suite containing four bedrooms and public areas. It is uncommon for a bedroom within a dorm suite to contain two or more beds and for students to have roommates.

Different from many Western universities, almost all universities in China provide on-campus dormitories for undergraduate students for their entire period of study, which is four years for most undergraduate programs[19-22]. Only a few students with special permission live off-campus. There are also substantial differences in the layouts of dorm rooms between the two. In the Chinese university dormitory, a typical dorm room contains a single bedroom with several beds, where each student occupies one bed and shares public areas in the dorm room with other roommates. In Supplementary Figure 1b, we show the floor plan of a typical Chinese university dorm room containing four beds and public areas including a balcony and washroom. The Chinese dorm room is much smaller than the dorm suite in Western universities, and it usually contains a larger number of beds, usually ranging from 2 beds to as many as over 20 beds in one single room, whereas 4 beds in a room for undergraduates are the most common setup in Chinese universities. This tight layout is in part due to limited resources but huge demands for on-campus accommodations. For example, at the university under study, there are about 5,000 students in each cohort (e.g., those who were admitted by the university in the same year) in the undergraduate programs, meaning that the university has to offer in total of about 20,000 beds for four cohorts of undergraduates. Dorm rooms in the same dormitory building are identical to each other. New students are randomly assigned to dorm rooms, and they live together for four years until graduation (see Section 1.2 for

details). Taken together, a Chinese dorm room provides a more interactive environment than a Western dorm suite, and it helps generate more interpersonal interactions between roommates.

Moreover, different from Western universities, where on-campus student residential buildings are integrated into local communities, most universities in China have clear geographical boundaries protected by fences, and university gates are guarded. In the university under study, dorm buildings are all within the university boundary (i.e., on campus), and they are identical and geographically very close (see Supplementary Figure 2 for a sketch of the campus layout). This suggests that dorm rooms should have the same or very similar environment.

1.2 Roommate assignment in university dorms

In Western countries, most universities provide on-campus dormitories to undergraduate students for the first year or the first six quarters of enrollment[14-18]. To live on campus, new students have to fill out housing applications to the university housing management office and sign a housing contract with the office. Throughout the application, students have the opportunity to choose a dormitory suite and provide their lifestyles and living preferences for consideration. The housing assignment team reviews each student's personal preferences and uses them for dorm and suite mate matching[18,23]. If students provide no such personal preferences, their dorm and suite mates will be randomly assigned[12,24-27]. Although not all personal preferences are guaranteed to be matched[28], the suitemate assignment is not completely random because students with similar lifestyles and living preferences have a higher probability of living together.

By contrast, in almost all Chinese universities, the assignment of roommates and dorm rooms is plausibly random[13,22,29-31]. Taking the university under study as an example, we consulted the university administrators from the Housing Management Office and the Student Admission Office, yielding three pieces of evidence supporting the random assignment.

First, the initial assignment followed university student accommodation management regulations, and there was no consideration of student personal preferences. Before student registration in the first semester, the housing office received a list of incoming students with the information of their name, gender, and student ID. Detailed departmental information is coded in the student ID (e.g., "2008060205003", a hypothetical student ID number to illustrate its structure; the same below), where the first four digits represent cohort (e.g., "2008", namely, the year of admission), each of the following two digits represents the school (e.g., "06": School of Computer Science and Engineering), major (e.g., "02": Information Security major in the school), and administrative unit (e.g., "05": the 5th unit in the major; one unit contains about 30 students), and the last three digits represent the student's order in their administrative unit (e.g., "003": the 3rd student in the unit). Upon receiving the student list, the housing office separated female and male students because there was no gender mix in dorm rooms or buildings and then assigned them to dorm rooms in order of student ID. There, students of the same gender, administrative unit, major, or school were assigned to the same 4-person dorm room, on the same floor, and in the same dorm building when possible. For example, students with student ID numbers "2008060205001," "2008060205002," "2008060205003," and "2008060205004" were assigned together. When an administrative unit had remaining students, they were paired with students of the same gender in the next unit, the same major, and the same school when possible. Our analysis of the accommodation data showed that the university's student accommodation management regulations were enforced on the initial roommate assignment of roommates. Specifically, we found that roommates tend to have nearby

student IDs (Supplementary Figure 3a), a pattern that is more pronounced in the actual data than in the roommate null model (see Section 3.1 for details), and roommates in most dorm rooms have the same major (Supplementary Figure 3b). Moreover, it was confirmed that the housing office neither had access to students' prior academic performance or other personal data nor allowed them to choose roommates or dorm rooms based on personal preferences. Students were informed of their accommodation information only when they checked in before the first semester.

Second, the initial assignment did not depend on the student's prior academic performance. We consulted the admission office to understand the relationship between student IDs and their academic performance before college enrollment. We were informed that there were no simple, one-size-fits-all criteria to compare the ability of students who were admitted into the same major. The assignment of student IDs was plausibly random for the following reasons. (1) The university used a quota system to admit students into different majors, and students were admitted in the order of majors to which they applied. The quotas for different provinces or regions were allocated according to the university's discretion and preferences, whereas the university-hosting province received more quotas. (2) The National Higher Education Entrance Examination (i.e., "Gaokao" in China) was held by provincial governments using different evaluation systems. The total scores of the exams were hardly comparable across different systems (not like the total SAT score in the US, for example). Therefore, no simple criteria were obvious enough for administrators to evaluate students' academic performance before college enrollment. (3) The admission office randomly assigned students in the same major to one or more administrative units (about 30 students in each administrative unit) and assigned a student ID number to each student (see above for details) without considering their prior academic performance. There was no mix of majors in the same administrative unit. For students in the same major, the only obvious consideration was gender. Specifically, in each administrative unit, male students were always assigned smaller student ID numbers (e.g., "2008060205003") than female students (e.g., "2008060205029").

Third, there was no significant selection process later in the semesters. Once assigned to a dorm room by the housing office, four students lived together until their graduation. There was no systematic reassignment of roommates or change of dorm rooms after the initial assignment. Moving out of a dorm room or from one dorm room to the other should be very rare on a few occasions. All applications for a dorm room change or roommate change were reviewed by the housing office. Exceptions were only given in justified circumstances, such as when a student transferred to a different program, applied for sick leave or had irreconcilable conflicts with other roommates. According to the housing office, these circumstances were very rare at the time.

Taken together, the initial assignment is plausibly random concerning students' prior academic performance and preferences. While we lacked data on student prior performance and personal data to directly verify the random assignment, our consultation with university administrators gave us extra confidence in assuming that the initial assignment of roommates and dorm rooms was plausibly random because the administrative processes involved no obvious consideration of student prior academic performance, socioeconomic background, or personal preference.

1.3 Student accommodation and academic performance data

From the university under study, we collect longitudinal data on both the accommodation and the academic performance of Chinese undergraduate students. The data covers the 2011 and 2012 cohorts of undergraduates, who were randomly assigned to identical 4-person dorm rooms before

the first semester and lived together until their graduation. There are no foreign students in the data. For accommodation, we collect each student's dorm room number. Students with the same dorm room number are identified as roommates, and this roommate relationship is static by construction. For academic performance, we collect the grade point average (GPA) for each student and semester in 2011-2014. The GPA data covers the first five consecutive semesters for the 2011 cohort and the first three consecutive semesters for the 2012 cohort. We exclude those students whose GPA information was not available at the time of data collection. For comparisons across different majors and cohorts, we transform each student's GPA into a percentile R among students in the same major and cohort for each semester, where $R = 0$ and $R = 1$ correspond to the lowest and highest academic performance, respectively. We inferred the major of each student from their student ID (see Section 1.2 for details). We dropped those for which we couldn't draw a confident inference or that had missing values in other variables. There are in total 36 majors for the two cohorts of students (34 majors for one cohort and 31 majors for the other). Most majors have 55~150 students, and majors with a few students are rare (Supplementary Figure 4). At the minimum, one major has fifteen students. Together, the data collection and cleaning process led to a sample of 5,272 students, who lived in identical 4-person dorm rooms for up to five semesters.

2. Tier combinations of roommate academic performance

To study roommate peer effects, we start by analyzing the composition of students in a typical 4-person dorm room in terms of their academic performance. Specifically, we divide students into equal-sized tiers based on their GPA percentiles R (hereafter GPA for simplicity) and compare the actual probability of observing a tier combination of roommates in the real data with its theoretical probability. In the following, we first introduce the theoretical probability of each tier combination under different tiers of classification (e.g., 3 tiers and 4 tiers). We then introduce the comparison between the actual and theoretical probabilities, followed by the empirical results for each semester.

2.1 The theoretical probability for each combination

Given a tier of classification, students are categorized into equal-sized tiers based on their GPA, where a larger tier number indicates better academic performance. For example, under the 2-tier classification, a student with $R = 0.7$ (i.e., GPA is higher than 70% of students in the same major) is in Tier 1. The tier number of the same student may change under different classifications. For example, the student is in Tier 3 under the 4-tier classification. Under a given tier of classification, each 4-person dorm room has a tier combination without particular order, for example, 3444 (i.e., one student is in Tier 3, and the other three students are in Tier 4) is identical to 4344, 4434, and 4443. Therefore, we use the one in ascending tier order to delegate all identical others. Under the 2-tier classification, there are five combinations (1111, 1112, 1122, 1222, and 2222). The numbers are 15 and 35 under the 3-tier and 4-tier classifications, respectively (see Supplementary Figure 5 for a list of all combinations under these classifications). Throughout the study, we only consider the setting of a typical 4-person dorm room (see Section 1.1 for details), for simplicity and consistency with the case of the university under study.

If there is no roommate peer effect, each roommate's GPA should be independent and identically distributed (i.i.d.), and the tier numbers of roommates determined by their GPA and the tier classification used should also be independent of each other. Therefore, we can calculate the theoretical probability P_t for each combination. It should be noted that the theoretical probabilities of different combinations are not always the same, and they shouldn't be directly compared with each other because the total numbers of identical combinations delegated by the combination in

ascending order are sometimes different. For a simple example, under the 2-tier classification, the theoretical probability P_t of combination 1112 is calculated by

$$P_t(1112) = C_4^1 \left(\frac{1}{2}\right)^3 \left(\frac{1}{2}\right) = \frac{1}{4}. \tag{1}$$

The value is four times as large as theoretical probability P_t of combination 1111 calculated by

$$P_t(1111) = \left(\frac{1}{2}\right)^4 = \frac{1}{16}. \tag{2}$$

The differences in the theoretical probabilities are because of combination 1112 delegates four identical combinations (namely, 1112, 1121, 1211, and 2111), while combination 1111 only delegates one identical combination. In Supplementary Table 1, we show all combinations and their theoretical probabilities under the 2-tier, 3-tier, and 4-tier classifications, respectively.

2.2 Comparison between actual and theoretical probabilities

The actual probability P_a of observing a combination in the actual data is calculated by the share of dorm rooms with the combination. If there are significant roommate peer effects on academic performance, the GPAs of roommates should be correlated, leading to a higher or lower probability of observing some combinations across all dorm rooms. As mentioned above, however, the actual probabilities of different combinations shouldn't be directly compared with each other because their theoretical probabilities are not always the same. This results in difficulties in assessing whether a combination is over-represented or under-represented in the real data only by looking at the face values of actual probabilities. To address this challenge, we introduce a metric named relative ratio $\mathbb{E}$, which is calculated by the relative difference between the actual probability and the theoretical probability of the same combination. Formally, the relative ratio $\mathbb{E}$ is given by

$$\mathbb{E} = \frac{P_a - P_t}{P_t}, \tag{3}$$

where P_a and P_t are the actual probability and the theoretical probability of the same combination, respectively. A positive (negative) relative ratio $\mathbb{E}$ means the combination is more (less) likely to be observed in the actual data than expected by chance. In the main text (Fig. 1), we have shown the relative ratio $\mathbb{E}$ of combinations under 2-tier, 3-tier, and 4-tier classifications for a 4-person dorm room based on the data for all five semesters together. Here we further show that the main conclusions are largely robust when using the data for each semester. Specifically, Supplementary Figure 5 presents all possible combinations in ascending order of tier numbers under 2-tier, 3-tier, and 4-tier classifications and the relative ratio $\mathbb{E}$ of each combination from semester 1 to semester 5, where each column corresponds to the data for one semester.

Analyzing the data, we notice that some combinations tend to have a larger relative ratio $\mathbb{E}$ than others, and this pattern appears to hold for all five semesters (Supplementary Figure 5). The result suggests that the actual probabilities of these combinations are larger than their theoretical probabilities. Moreover, we find that these combinations tend to contain nearby tier numbers such as 1111, and 1112, suggesting that roommates tend to have similar academic performance. This observation prompts us to further explore the relationship between the tier heterogeneity of a combination (i.e., the differences in the tier numbers) and its relative ratio $\mathbb{E}$. More specifically, we first calculate the relative difference D in the tier numbers of each combination:

$$D = \frac{1}{6}\sum_{u \neq v}|l_u - l_v|, 1 \leq u < v \leq 4, \tag{4}$$

where l_u and l_v is the tier number of roommates u and v, respectively. A smaller relative difference D means that roommates have closer tier numbers and thus more similar academic

performance. In Supplementary Figure 6, we group combinations with the same D and rearrange the order of combinations in the descending order of D and tier numbers. We find that there appears to be a negative association between the relative ratio $\mathbb{E}$ and the relative difference D, where $\mathbb{E}$ is larger for combinations with smaller D. This pattern is more pronounced when we take an average of $\mathbb{E}$ for all combinations with the same D. Specifically, Supplementary Figure 7 presents a much clear positive association between $\mathbb{E}$ and D for each of the five semesters. This result suggests a higher probability of observing a dorm room in which roommates have smaller differences in their academic performance in the actual data than its theoretical value. Together, these results show that roommates are more similar in academic performance, suggesting the presence of roommate peer effects (see Section 3.2 for detailed statistical testing results).

3. Roommate null model and statistical test

We perform a statistical hypothesis test to further demonstrate the presence of roommate peer effects on academic performance by comparing observations from the actual data with those from a roommate null model. In the following, we first introduce the method for constructing the roommate null model based on the actual accommodation data. We then introduce the method for the statistical hypothesis testing and the results for quantifying roommate peer effects.

3.1 Construction of a roommate null model

The null model is a widely used approach to studying non-trivial features of complex systems[32-34]. An appropriate null model of a complex system not only satisfies a collection of constraints, for instance, a network null model that preserves degree distributions of the original network[35-37], but also provides an unbiased random structure of other features, offering a baseline to examine whether displayed features of interest in the original complex system are truly non-trivial. Over the past few decades, null-model approaches have been applied to test causal effects in many complex social systems[38-40]. For instance, in the social network literature, randomizations as one type of null models are used to study the impact of network interventions on social relationships[41]. Building on traditional statistical methods and inspired by recent applications of null-model approaches, we construct a roommate null model to test whether roommates are more similar in their academic performance than expected by chance alone.

Ideally, we would want the roommate null model to replicate the actual random assignment of roommates, and at the same time, it takes into account any non-random aspects of the actual assignment (see Section 1.2 for details). To build a roommate null model, we start with the actual roommate configuration in the real data and randomly shuffle students between dorm rooms while preserving their compositions of cohort, gender, and major. Specifically, in practice, each time we randomly select two students in the same cohort, gender, and major and then swap them into each other's dorm room. Besides these controlled characteristics during the random shuffling process, the two students should have random roommates concerning their academic performance and other characteristics. We repeat the student random selection and swapping many times, for example, over 100,000 times to produce one implementation. By repeating this entire process, we construct a plausible roommate null model that consists of 1000 independent implementations. We show that the roommate null model that we construct successfully preserves these controlled student characteristics. For example, the major compositions of all dorm rooms in the roommate null model are the same as in the actual data (Supplementary Figure 3b).

3.2 Statistical hypothesis testing

A statistical test is a powerful tool for examining whether the empirical data sufficiently supports a particular hypothesis that is tested. In the main text (Fig. 1), we find that the relative ratio $\mathbb{E}$ of different combinations varies and deviates from 0. For example, $\mathbb{E}$ of combination 1111 is positive and much larger than others such as combination 1122. Here we further perform a statistical test to examine whether a combination's actual relative ratio $\mathbb{E}$ deviates significantly from its theoretical value which is 0, in other words, whether the actual probability P_a is significantly different from its theoretical probability P_t. Considering that P_t of a combination is only one value, making it hard to tell whether P_a is significantly larger or smaller, we employ the roommate null model to perform a statistical test, where the null-model probability P_n is used to proxy P_t.

Specifically, given a tier of classification, we first generate a roommate null model (see Section 3.1 for details) and calculate the null-model relative ratio for each combination:

$$\widetilde{\mathbb{E}} = \frac{P_n - P_t}{P_t}, \tag{5}$$

where P_n and P_t are the combination's null-model and theoretical probabilities, respectively. By the null-model construction, P_n should approach P_t, and thus $\widetilde{\mathbb{E}}$ should be close to 0. For each combination, we compare the actual E with its null-model $\widetilde{\mathbb{E}}$. If $\mathbb{E}$ is significantly above 0, the probability of observing $\mathbb{E} \leq \widetilde{\mathbb{E}}$ in the actual data should be sufficiently small, e.g., less than 0.001. Accordingly, our null hypothesis (H0) is $\mathbb{E} \leq \widetilde{\mathbb{E}}$, and the alternative hypothesis (H1) is $\mathbb{E} > \widetilde{\mathbb{E}}$. To empirically test H0, we generate 1000 roommate null models (where each null model is an independent implementation of the random shuffling process) and calculate $\widetilde{\mathbb{E}}$ under 2-tier, 3-tier, and 4-tier classifications, respectively. We find that $\mathbb{E}$ of some combinations is larger than $\widetilde{\mathbb{E}}$ for all roommate null models, allowing us to reject H0 and support H1 (i.e., $\mathbb{E}$ is significantly larger than 0 with a P value < 0.001 in the one-sided statistical test; the combination is over-represented in the actual data). Similarly, we test whether $\mathbb{E}$ of a combination is significantly below 0. Under the 2-tier classification, for example, combinations with significantly positive $\mathbb{E}$ include 1111 and 2222 (P value < 0.001) and those with significantly negative $\mathbb{E}$ include 1112 and 1122 (P value < 0.001) as well as 1222 (P value < 0.05). In Supplementary Table 2, we show the statistical testing results for each combination under 2-tier, 3-tier, and 4-tier classifications, respectively.

To perform a single statistical test for all combinations together given a tier of classification, we calculate the total relative ratio $\sum|\mathbb{E}|$ and $\sum|\widetilde{\mathbb{E}}|$ by summing up the absolute $\mathbb{E}$ and $\widetilde{\mathbb{E}}$ of each combination, respectively. As $\widetilde{\mathbb{E}}$ is close to 0, $\sum|\widetilde{\mathbb{E}}|$ should also be close to 0. If we assume $\sum|\mathbb{E}| \leq \sum|\widetilde{\mathbb{E}}|$, it is naturally that $\sum|\mathbb{E}|$ is close to 0, yielding $\mathbb{E}$ to be close to 0. There, $\mathbb{E}$ and $\widetilde{\mathbb{E}}$ wouldn't have a significant difference because they are both close to 0. Thereby, to say $\mathbb{E}$ is significantly different from $\widetilde{\mathbb{E}}$, the probability of observing $\sum|\mathbb{E}| \leq \sum|\widetilde{\mathbb{E}}|$ should be sufficiently small, e.g., less than 0.001. Accordingly, our null hypothesis (H0) is $\sum|\mathbb{E}| \leq \sum|\widetilde{\mathbb{E}}|$, and the alternative hypothesis (H1) is $\sum|\mathbb{E}| > \sum|\widetilde{\mathbb{E}}|$. We find that, under 2-tier, 3-tier, and 4-tier classifications, $\sum|\mathbb{E}|$ is always larger than $\sum|\widetilde{\mathbb{E}}|$ for 1000 roommate null models, allowing us to reject H0 and support H1 with a P value < 0.001 (i.e., the overall $\mathbb{E}$ of all combinations is different from 0). Taken together, our hypothesis testing results show that $\mathbb{E}$ of some tier combinations in the actual data deviates significantly from 0, where those with nearby tier numbers of academic performance are more likely and those with distant tier numbers are less likely to be observed than random chance, suggesting that roommate peer effects on academic performance are significant.

4. Assimilation of roommate academic performance

The positive association between the relative ratio $\mathbb{E}$ and the relative difference D of student tier combinations in dorm rooms suggests that roommates with similar academic performance are more likely to be observed in the real data than expected by chance. To examine this relationship at a more granular level, we develop an assimilation metric to measure the differences in roommate academic performance. In the following, we first introduce the assimilation metric and then introduce the statistical test to demonstrate the presence of roommate peer effects.

4.1 Academic performance assimilation

We generalize the analysis of tier combinations to the most granular tier for classification by directly dealing with the original GPA percentile $R \in [0, 1]$ (hereafter, GPA for short), where a larger value means better academic performance. More specifically, we develop an assimilation metric A to quantify the differences in the GPAs between roommates (Supplementary Figure 8). Formally, the assimilation A for a 4-person dorm room is calculated by

$$A = 1 - \frac{1}{4}\sum_{u \neq v}|R_u - R_v|, 1 \leq u < v \leq 4, \tag{6}$$

where R_u and R_v are the GPAs of roommates u and v, respectively. The assimilation A is between 0 and 1, where a larger value means that roommates have more similar academic performance. Based on the data, we can calculate the distribution of actual assimilation A of all dorm rooms. If there is no roommate peer effect, the R of each roommate should be independent and identically distributed (i.i.d.), and the value of R follows a uniform distribution in [0, 1]. Therefore, we can calculate the expectation (mean value) of the theoretical assimilation A_t for a 4-person dorm room:

$$\langle A_t \rangle = 1 - \frac{3}{2} \times \iint_0^1 |x - y|\, dx\, dy = \frac{1}{2}. \tag{7}$$

To perform a statistical test on whether the actual assimilation is significantly larger than its theoretical value, we use the null-model assimilation that is calculated based on the roommate null model (see Section 3.1 for details) to proxy the theoretical assimilation.

We compare the distribution of the actual assimilation for all dorm rooms with the one under the roommate null model that consists of 1000 independent implementations. For all five semesters together (see Fig. 2a in the main text), we find that the mean actual assimilation in the actual data is 0.549, which is much larger than the mean null-model assimilation in the null model (0.496). When looking at each semester (Supplementary Figure 8a), we find that the main result holds robustly and the mean actual assimilation exhibits an overall increasing trend. Specifically, the mean actual assimilation has steadily increased in the first three semesters (Supplementary Figure 8b), which is consistent with the literature showing that peer effects are strong and persistent when student friendships or peer relationships last over a year[21,42,43], but starts to decrease in the fifth semester, which may be because that senior undergraduates can take different elective courses and have more outside activities that decrease their interactions with roommates[44].

4.2 Statistical tests for differences in assimilation

We perform a statistical test on whether the actual assimilation is significantly different from the null-model assimilation. Specifically, we use the student's t-test, which is a common application to test whether the means of two distributions are significantly different. Our null hypothesis (H0) is that the two assimilation distributions are from the same distribution. The student's t-test performed on the actual data rejects the null hypothesis (H0) as the P value is less than 0.001. The

result supports that the null-model assimilation and the actual assimilation are from different distributions, and they have different means (student's t-test; P value < 0.001). Specifically, the mean actual assimilation (0.549) of all dorm rooms is about 10.7% larger than the mean null-model assimilation (0.496; Supplementary Figure 8b), which is very close to its theoretical value of 0.5 in complete randomization. Taken together, these results suggest that roommates exhibit larger assimilation than random chance, demonstrating the presence of roommate peer effects.

4.3 Robustness check for the assimilation results

In the main analysis, we compare the means of the actual assimilation distribution with the null-model assimilation distribution, where the student's t-test suggests that the two distributions are different. Here we check the robustness of these results using an alternative approach, where we examine the significance of each dorm room's assimilation. Specifically, we first calculate the average assimilation (mean) of all dorm rooms under the roommate null model consisting of 100 independent implementations (see Section 3.1 for details) and its standard error (*s.e.*). We then calculate the assimilation of each dorm room and identify those that have significantly larger assimilation than the average null-model assimilation (P value = 0.05; a value that is larger than mean+1.96×*s.e.*, i.e., the upper 95% confidence interval). We find that about 60% of dorm rooms have larger-than-null-model assimilation (Supplementary Figure 9a), and this fraction exhibits an increasing trend as well (Supplementary Figure 9b), suggesting that peer effects overall increase as students live together longer. Our conclusions are supported by two different methods.

4.4 The assimilation results by different gender

In the university under study, female and male students were assigned to dorm rooms in different buildings, and there was no gender mix in dorm rooms or buildings. This motivates us to unpack the assimilation analysis by gender and add gender as a control to the roommate null model and the OLS regression model. We find that while female students represent only a small proportion of the student sample (about 23%), they have significantly better academic performance on average than their male counterparts (P value < 0.001; Supplementary Figure 10a), which is consistent with the positive coefficient of the gender dummy (where females are in the treatment group) in Table 1 and Table 2 of the main text. Yet, here we find that there is no significant gender difference in the average assimilation either for all five semesters together (P value = 0.18; Supplementary Figure 10b) or for each semester (P value > 0.1; Supplementary Figure 10c), suggesting that roommate peer effects are prevalent across genders.

5. Regression models and regression results

We perform a Granger causality type of analysis to better understand roommate peer effects. Briefly, we use an ordinary least squares (OLS) model to regress a student's GPA in the current semester (GPA_Post) against several different factors including the student's GPA in the previous semester (GPA_Prior), the roommate prior average GPA (RM_Avg), the differences in roommate prior GPAs (RM_Diff), the student's in-dorm ordinal rank according to prior GPA (OR_InDorm), and the dummy variables of the student cohort, gender, major, and semester. Supplementary Table 3 shows the summary statistics of all variables and their symbols. All independent variables are mean-centered before being included in the OLS regression except for dummies.

5.1 Regression on the effects of peer heterogeneity

In our first analysis, we employ an OLS model to regress the relationship between a student's future GPA (GPA_Post) and the roommates' average prior GPA (RM_Avg). As we are interested

in the effects of peer heterogeneity, we include the differences in the roommates' prior GPAs (RM_Diff) and their interaction with RM_Avg in the OLS model. Other factors may independently affect a student's future GPA. Therefore, we include controls on the student's prior GPA, cohort, gender, major, and semester. Specifically, the empirical specification is given by

$$G_i^{s+1} = b_0 + b_1 G_i^s + b_2 RA_i^s + b_3 RD_i^s + b_4 RA_i^s \times RD_i^s$$
$$+ b_5 D^{Ge} + b_6 D^{Ma} + b_7 D^{Co} + b_8 D^{Se} + \epsilon_i, \quad (8)$$

where ϵ_i is the error term for student i, and the semester index s ranges from 1 to 4. The dependent variable G_i^{s+1} is the student's GPA in semester s+1 (GPA_Post), and the independent variable of interest G_i^s is the student's GPA in semester s (GPA_Prior), respectively. The variable RA_i^s is the average GPA of roommates in semester s (RM_Avg), RD_i^s is the differences in roommate prior GPAs (RM_Diff), and $RA_i^s \times RD_i^s$ is their interaction term. The variable D^{Ge} is gender dummy, which is coded as 1 and 0 for female and male students, respectively. The variables D^{Ma}, D^{Co}, and D^{Se} are major, cohort, and semester dummies, respectively.

The regression results for all five semesters together are summarized in Table 1 of the main text. We find that (1) the average prior GPA of roommates has a significantly positive effect on a student's post-GPA, (2) a student's prior GPA has the strongest effect on a student's post-GPA, and (3) the differences in roommate prior GPAs moderates the relationship between post-GPA and roommate average prior GPA such that the positive effects of roommate average prior GPA on a student's post-GPA are more pronounced when the differences in roommate prior GPAs are small. Fig. 3d of the main text shows the margin plot of the linear relationship between GPA_Post and RM_Avg, which is moderated by RM_Diff. To better assess the significance of the moderating effect, we perform a simple slope testing and summarize the results in Supplementary Figure 11a. We find that the slope decreases slightly with the increase of RM_Diff but it remains positive and statistically different from 0 (P value < 0.1). More specifically, the slope b = 0.055 (*s.e.* = 0.008; 95% CI = [0.040, 0.070]) when the differences in roommate prior GPAs are low (i.e., 1 *s.d.* below its mean), and the slope b = 0.028 (*s.e.* = 0.015; 95% CI = [−0.001, 0.057]) when the differences in roommate prior GPA are high (i.e., 1 *s.d.* above its mean). These results show that the positive relationship between a student's post-GPA and their roommate average prior GPA is more (less) pronounced when the differences in their roommate prior GPAs are low (high), suggesting the significant moderating role of peer heterogeneity on roommate peer effects.

While the regression analysis suggests that the roommate peer effects are significant, the effect size appears to be modest. Specifically, a 100-point increase in the roommate average prior GPA is associated with a 5-point increase in the student's GPA (see Table 1 in the main text). The effect is about 6% as large as the effect of a 100-point increase in the focal student's prior GPA, and it is about 10% of the student's own GPA. We reviewed the literature and found that prior studies reported effect sizes at a similar scale for various environments (e.g., dormitories and classrooms) and cultures (e.g., Western universities). For instance, Sacerdote (2001) found that a 1-*s.d.* increase in the roommate's GPA is associated with a 0.05 increase in the student's freshmen year GPA (an effect size of about 4% of their own GPA)[12], Zimmerman (2003) found that a 100-point increase in roommate verbal SAT score translated into a 0.03 increase in the student's first-semester GPA (an effect size of about 15% as large as a 100-point increase in their own verbal SAT score)[24], Carman et al. (2012) found that a 0.1-*s.d.* increase in peer average math achievement increases the student's math test score by 0.04 *s.d.* on average[9], and Thiemann (2021) reported negative results that 1-*s.d.* increase in average peer ability is associated with a decrease in a student's GPA by 7.9%

of 1-*s.d.* on average[45]. Presumedly, other external factors may contribute to this observation, such as that dorm rooms are not formal learning environments like classrooms and libraries, and there are other channels through which students can learn from and influence each other.

5.2 Regression on the effects of in-dorm ordinal rank

Competitive dynamics between roommates in the interpersonal and local dorm room environment could also affect student academic performance[46-48]. For example, on one extreme, in a dorm room that contains four students all with high academic performance (e.g., GPA >= 0.9), there is a possibility that one student's ordinal rank in the dorm room is always 4th across different semesters (e.g., GPA = 0.9, which is smaller than the GPAs of their three roommates). In this case, even if the student has high academic performance, they may still feel discouraged and less motivated due to their consistently lowest relative academic performance in the dorm room. On the other extreme, one student's ordinal rank in the dorm room is always 1st, conditional on absolute academic performance, they may feel more confident than others and have better long-term outcomes[49-51]. This prompts us to study how a student's post-GPA is impacted by their in-dorm ordinal rank according to prior GPA (OR_InDorm), which is the number of better-achieving roommates including themselves, with 1 being the highest and 4 being the lowest in performance.

Specifically, in the analysis, we employ an OLS model to study how a student's in-dorm ordinal rank (OR_InDorm) affects their future performance (GPA_Post) after controlling their prior performance, the average and differences in roommate prior performance, their gender, major, cohort, and semester. Meanwhile, we examine how peer heterogeneity in prior performance moderates this effect. Specifically, the empirical specification is given by

$$G_i^{s+1} = b_0 + b_1 G_i^s + b_2 OR_i^s + b_3 RA_i^s + b_4 RD_i^s + b_5 OR_i^s \times G_i^s + b_6 OR_i^s \times RA_i^s$$
$$+ b_7 OR_i^s \times RD_i^s + b_8 D^{Ge} + b_9 D^{Ma} + b_{10} D^{Co} + b_{11} D^{Se} + \epsilon_i, \quad (9)$$

where OR_i^s is the OR_InDorm of student i in semester s (ranging from 1 to 4) and ϵ_i is the error term. The interaction terms are $OR_i^s \times G_i^s$ between OR_InDorm and GPA_Prior, $OR_i^s \times RA_i^s$ between OR_InDorm and RM_Avg, and $OR_i^s \times RD_i^s$ between OR_InDorm and RM_Diff for student i in semester s. All other controls are the same as introduced above.

The regression results for all five semesters are summarized in Table 2 of the main text. We find that (1) in-dorm ordinal rank has positive effects on post-GPA, (2) neither the interaction term of in-dorm ordinal rank and prior GPA nor the interaction term of in-dorm ordinal rank and roommate average prior GPA is significant, and (3) the interaction term of in-dorm ordinal rank and the differences in roommate prior GPA is significantly negative. The results suggest the significant moderating role of peer heterogeneity. In Fig. 4b of the main text, we show the margin plot of the linear relationship between GPA_Post and OR_InDorm, which is moderated by RM_Diff. To assess the significance of the moderating effect of RM_Diff on the relationship, we perform a simple slope testing and summarize the results in Supplementary Figure 11b. We find that the slope decreases with the increase of RM_Diff and becomes not statistically different from 0 when RM_Diff > −0.3 *s.d.* (*P* value > 0.1). In particular, the slope *b* = 0.007 (*s.e.* = 0.003; 95% CI = [0.002, 0.012]; *P* value = 0.009) when RM_Diff is low (i.e., 1 *s.d.* below its mean), and the slope *b* = −0.000 (*s.e.* = 0.004; 95% CI = [−0.007, 0.007]; *P* value = 0.982) when RM_Diff is high (i.e., 1 *s.d.* above its mean). These results suggest that the positive relationship between a student's post-GPA and their roommate average prior GPA is more pronounced when the differences in their roommate prior GPA are low but loses significance when the differences are high.

5.3 Falsification tests of the regression results

To check the robustness of the OLS regression results and demonstrate significance, we conduct a falsification test by running the same OLS regressions on the roommate null model that consists of 100 independent implementations (see Section 3.1 for details). In the null model, students are assumed to live together for all semesters once assigned to the same room. The falsification testing results are summarized in Supplementary Figure 12 and Supplementary Figure 13.

Analyzing the OLS regression results based on the roommate null model, we find that the effect of roommate average prior performance (RM_Avg) on a student's future performance (GPA_Post) is very small (b = 0.004 in the null model vs 0.050 in the actual data, both with P value < 0.01), and the moderation effect of the differences in roommate prior performance (RM_Diff) is also very small (b = –0.011 in the null model vs –0.089 in the actual data, both with P value < 0.1; see Supplementary Figure 12 and Fig. 3 of the main text). Although simple slope testing results show that, in the roommate null model, the effect of RM_Avg on GPA_Post is more pronounced and significant (b = 0.004, P value < 0.01) when RM_Diff is low (i.e., 1 *s.d.* below its mean; Supplementary Figure 14a), the effect is still much smaller than that in the actual data (b = 0.066, P value < 0.01; Supplementary Figure 11a). Together, these results show that the effect size of RM_Avg and RM_Diff on GPA_Post in the roommate null model is only about 10% of that in the actual data, which suggests the significance of the findings from the regression analysis.

Moreover, we find that the effect of a student's within-dorm ordinal rank according to prior performance (OR_InDorm) on the student's future performance (GPA_Post) is very small (b = 0.001 in the null model vs 0.006 in the actual data; both with P value < 0.05), and the moderating effect of RM_Diff is also very small (b = –0.007 in the null model with P value < 0.01 vs –0.022 in the actual data with P value < 0.05; see Supplementary Figure 13 and Fig. 4 of the main text). These results show that the effect size of OR_InDorm and RM_Diff on GPA_Post in the null model is about 20% of that in the actual data. Interestingly, we notice that the relationship between OR_InDorm and GPA_Post is significantly negative in the null model (b = –0.0013, P value < 0.01) when RM_Diff is high (i.e., 1 *s.d.* above its mean; see Supplementary Figure 13c and Supplementary Figure 14b). This is because when RM_Diff is high, the student can only have a high GPA_Prior when their OR_InDorm is low (e.g., ranked 1st in the dorm room) and a low GPA_Prior when their OR_InDorm is high (e.g., ranked 4th in the dorm room). There, the high correlation between GPA_Prior and GPA_Post leads to a moderate negative relationship between OR_InDorm and GPA_Post. The significantly positive relationship between OR_InDorm and GPA_Post in the null model (b = 0.0008, P value < 0.01) when RM_Diff is low (i.e., 1 *s.d.* below its mean) is, however, due to "regression toward the mean" when all dorm rooms are included in the regression (Supplementary Figure 13c and Supplementary Figure 14b).

Taken together, these results from the falsification tests on the roommate null model support our findings that roommate peer effects on academic performance are nontrivial and significant although the magnitude of the effects appears to be not large (see Section 5.1 for details).

Supplementary References

1 Schudde, L. T. The causal effect of campus residency on college student retention. *Review of Higher Education* **34**, 581-610 (2011).

2 Lau, E. Y. Y., Wong, M. L., Ng, E. C. W., Hui, C.-c. H., Cheung, S. F. & Mok, D. S. Y. "Social jetlag" in morning-type college students living on campus: Implications for physical and psychological well-being. *Chronobiology International* **30**, 910-918 (2013).

3 Ong, S.-E., Petrova, M. & Spieler, A. Demand for university student housing: An empirical analysis. *Journal of Housing Research* **22**, 141-164 (2013).

4 Graham, P. A., Hurtado, S. S. & Gonyea, R. M. The benefits of living on campus: Do residence halls provide distinctive environments of engagement? *Journal of Student Affairs Research and Practice* **55**, 255-269 (2018).

5 Simpson, D. B. & Burnett, D. Commuters versus residents: The effects of living arrangement and student engagement on academic performance. *Journal of College Student Retention: Research, Theory & Practice* **21**, 286-304 (2019).

6 Garvey, J. C., Ballysingh, T. A., Dow, L. B., Howard, B. L., Ingram, A. N. & Carlson, M. Where I sleep : The relationship with residential environments and first-generation belongingness. *College Student Affairs Journal* **38**, 16-33 (2020).

7 Cao, Y., Gao, J., Lian, D., Rong, Z., Shi, J., Wang, Q., Wu, Y., Yao, H. & Zhou, T. Orderliness predicts academic performance: Behavioural analysis on campus lifestyle. *Journal of The Royal Society Interface* **15**, 20180210 (2018).

8 Hoxby, C. M. Peer effects in the classroom: Learning from gender and race variation. (2000).

9 Carman, K. G. & Zhang, L. Classroom peer effects and academic achievement: Evidence from a Chinese middle school. *China Economic Review* **23**, 223-237 (2012).

10 Lavy, V., Paserman, M. D. & Schlosser, A. Inside the black box of ability peer effects: Evidence from variation in the proportion of low achievers in the classroom. *Economic Journal* **122**, 208-237 (2012).

11 Burke, M. A. & Sass, T. R. Classroom peer effects and student achievement. *Journal of Labor Economics* **31**, 51-82 (2013).

12 Sacerdote, B. Peer effects with random assignment: Results for Dartmouth roommates. *Quarterly Journal of Economics* **116**, 681-704 (2001).

13 Zhang, L. & Pu, S. It takes two shining lights to brighten the room: Peer effects with random roommate assignments. *Education Economics* **25**, 3-21 (2017).

14 Strother, L., Piston, S., Golberstein, E., Gollust, S. E. & Eisenberg, D. College roommates have a modest but significant influence on each other's political ideology. *Proceedings of the National Academy of Sciences, U.S.A.* **118**, e2015514117 (2021).

15 Lopez Turley, R. N. & Wodtke, G. College residence and academic performance: Who benefits from living on campus? *Urban Education* **45**, 506-532 (2010).

16 La Roche, C. R., Flanigan, M. A. & Copeland Jr, P. K. Student housing: Trends, preferences and needs. *Contemporary Issues in Education Research* **3**, 45-50 (2010).

17 Yanni, C. *Living on campus: An architectural history of the American dormitory*. (2019).

18 The University of Chicago. *New student housing assignments*, <https://college.uchicago.edu/housing-residence-life/new-student-housing-assignments> (2023).

19 Xu, M., de Bakker, M., Strijker, D. & Wu, H. Effects of distance from home to campus on undergraduate place attachment and university experience in China. *Journal of Environmental Psychology* **43**, 95-104 (2015).

20 Yao, H., Lian, D., Cao, Y., Wu, Y. & Zhou, T. Predicting academic performance for college students: A campus behavior perspective. *ACM Transactions on Intelligent Systems and Technology* **10**, 24 (2019).

21 Cheng, C. Duration matters: Peer effects on academic achievement with random assignment in the Chinese context. *Journal of Chinese Sociology* **7**, 2 (2020).

22 Ma, L. & Huang, Y. Roommate effects on students' performance in higher education. *Peking University Education Review* **19**, 41-63 (2021).

23 Eisenberg, D., Golberstein, E. & Whitlock, J. L. Peer effects on risky behaviors: New evidence from college roommate assignments. *Journal of Health Economics* **33**, 126-138 (2014).

24 Zimmerman, D. J. Peer effects in academic outcomes: Evidence from a natural experiment. *Review of Economics and Statistics* **85**, 9-23 (2003).

25 McEwan, P. J. & Soderberg, K. A. Roommate effects on grades: Evidence from first-year housing assignments. *Research in Higher Education* **47**, 347-370 (2006).

26 Guo, G., Li, Y., Owen, C., Wang, H. & Duncan, G. J. A natural experiment of peer influences on youth alcohol use. *Social Science Research* **52**, 193-207 (2015).

27 Babcock, P., Bedard, K., Fischer, S. & Hartman, J. Coordination and contagion: Individual connections and peer mechanisms in a randomized field experiment. *Journal of Public Economics* **185**, 104069 (2020).

28 Chen, Y. & Snmez, T. Improving efficiency of on-campus housing: An experimental study. *American Economic Review* **92**, 1669-1686 (2002).

29 Han, L. & Li, T. The gender difference of peer influence in higher education. *Economics of Education review* **28**, 129-134 (2009).

30 Pu, S., Yan, Y. & Zhang, L. Peers, study effort, and academic performance in college education: Evidence from randomly assigned roommates in a flipped classroom. *Research in Higher Education* **61**, 248-269 (2020).

31 Pu, S., Yan, Y. & Zhang, L. Do peers affect undergraduates' decisions to switch majors? *Educational Researcher* **50**, 516-526 (2021).

32 Mucha, P. J., Richardson, T., Macon, K., Porter, M. A. & Onnela, J.-P. Community structure in time-dependent, multiscale, and multiplex networks. *Science* **328**, 876-878 (2010).

33 Cimini, G., Squartini, T., Saracco, F., Garlaschelli, D., Gabrielli, A. & Caldarelli, G. The statistical physics of real-world networks. *Nature Reviews Physics* **1**, 58-71 (2019).

34 Váša, F. & Mišić, B. Null models in network neuroscience. *Nature Reviews Neuroscience* **23**, 493-504 (2022).

35 Maslov, S. & Sneppen, K. Specificity and stability in topology of protein networks. *Science* **296**, 910-913 (2002).

36 Milo, R., Shen-Orr, S., Itzkovitz, S., Kashtan, N., Chklovskii, D. & Alon, U. Network motifs: Simple building blocks of complex networks. *Science* **298**, 824-827 (2002).

37 Smart, A. G., Amaral, L. A. N. & Ottino, J. M. Cascading failure and robustness in metabolic networks. *Proceedings of the National Academy of Sciences, U.S.A.* **105**, 13223-13228 (2008).

38 Krackhardt, D. Predicting with networks: Nonparametric multiple regression analysis of dyadic data. *Soc Networks* **10**, 359-381 (1988).

39 Hubert, L. & Schultz, J. Quadratic assignment as a general data analysis strategy. *Brit J Math Stat Psy* **29**, 190-241 (1976).

40 Raudenbush, S. W. & Bryk, A. S. *Hierarchical linear models: Applications and data analysis methods*. Vol. 1 (Sage Publications, 2002).

41 Boda, Z., Elmer, T., Vörös, A. & Stadtfeld, C. Short-term and long-term effects of a social network intervention on friendships among university students. *Scientific Reports* **10**, 2889 (2020).

42 Foster, G. It's not your peers, and it's not your friends: Some progress toward understanding the educational peer effect mechanism. *Journal of Public Economics* **90**, 1455-1475 (2006).

43 Patacchini, E., Rainone, E. & Zenou, Y. Heterogeneous peer effects in education. *Journal of Economic Behavior & Organization* **134**, 190-227 (2017).

44 Deslauriers, L., McCarty, L. S., Miller, K., Callaghan, K. & Kestin, G. Measuring actual learning versus feeling of learning in response to being actively engaged in the classroom. *Proceedings of the National Academy of Sciences, U.S.A.* **116**, 19251-19257 (2019).

45 Thiemann, P. The persistent effects of short-term peer groups on performance: Evidence from a natural experiment in higher education. *Management Science* **68**, 1131-1148 (2022).

46 Bahns, A. J., Crandall, C. S., Canevello, A. & Crocker, J. Deciding to dissolve: Individual-and relationship-level predictors of roommate breakup. *Basic and Applied Social Psychology* **35**, 164-175 (2013).

47 Chakravarti, A., Menon, T. & Winship, C. Contact and group structure: A natural experiment of interracial college roommate groups. *Organization Science* **25**, 1216-1233 (2014).

48 Yu, H. Am I the big fish? The effect of ordinal rank on student academic performance in middle school. *Journal of Economic Behavior & Organization* **176**, 18-41 (2020).

49 Denning, J. T., Murphy, R. & Weinhardt, F. Class rank and long-run outcomes. *Review of Economics and Statistics* **105**, 1426-1441 (2023).

50 Murphy, R. & Weinhardt, F. Top of the class: The importance of ordinal rank. *Review of Economic Studies* **87**, 2777-2826 (2020).

51 Kinsler, J. & Pavan, R. Local distortions in parental beliefs over child skill. *Journal of Political Economy* **129**, 81-100 (2021).

Supplementary Figures

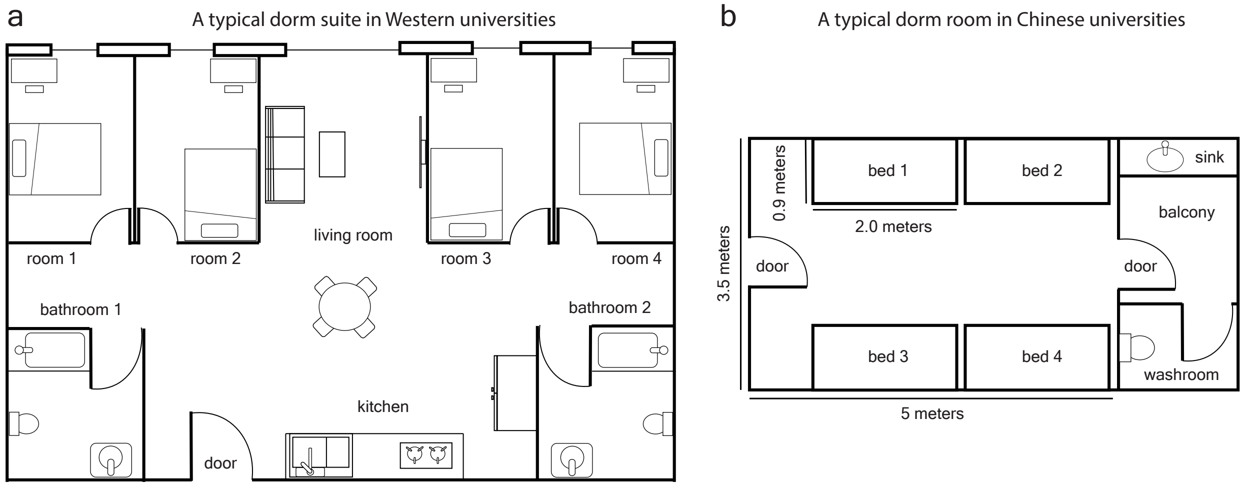

Supplementary Figure 1. Examples of dorm suites in Western universities and dorm rooms in Chinese universities. (a) Floor plan of a typical dorm suite in Western universities. A 4-person dorm suite has four separate bedrooms, where each student occupies one bedroom and shares public areas with suitemates such as a living room, kitchen, and bathroom. The dorm suite offers a private and independent space for each student. (b) Floor plan of a typical dorm room in Chinese universities. A 4-person dorm room contains four beds, where each student occupies one bed and shares public areas with roommates such as a balcony and washroom. The dorm room provides a small but interpersonal space for all roommates to frequently interact with each other.

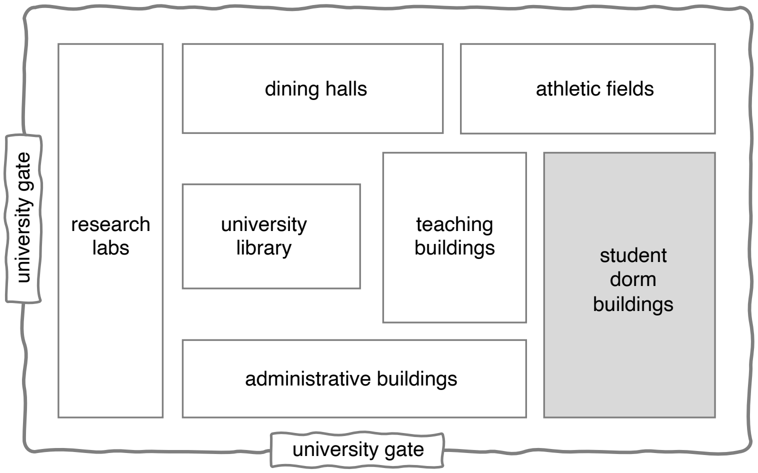

Supplementary Figure 2. A sketch of the campus facility layout of the university under study. The university campus has clear geographical boundaries protected by fences, and gates are guarded for security reasons. Dorm buildings are all within the university's geographic boundary (i.e., on campus), and they are identical in physical environment and very close to each other in distance.

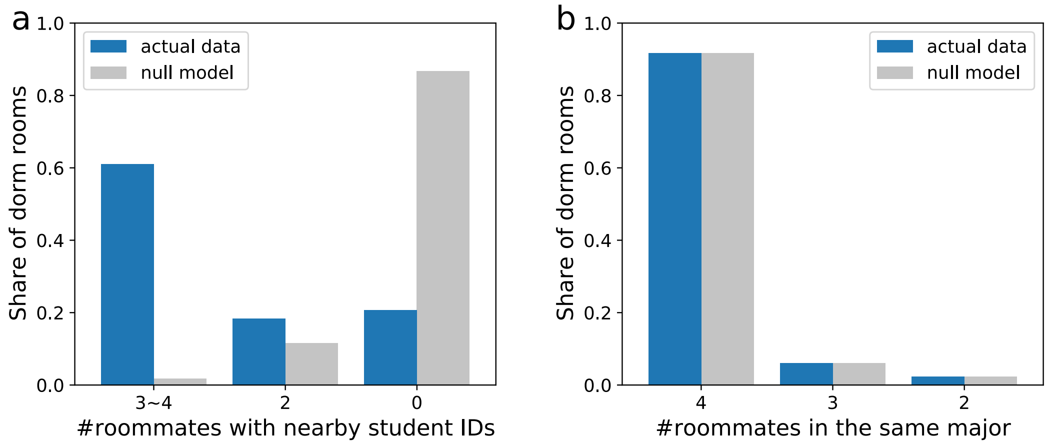

Supplementary Figure 3. Some patterns of the initial roommate assignment. (a) Roommates tend to have nearby student IDs. In more than 60% of dorm rooms, at least three roommates have nearby student IDs in the actual data. By comparison, this fraction is less than 2% in the roommate null model. (b) Roommates tend to have the same major. In more than 90% of dorm rooms, four roommates have the same major. The results are the same in the actual data and the roommate null model because the null model controls for the student's major in the random shuffling process.

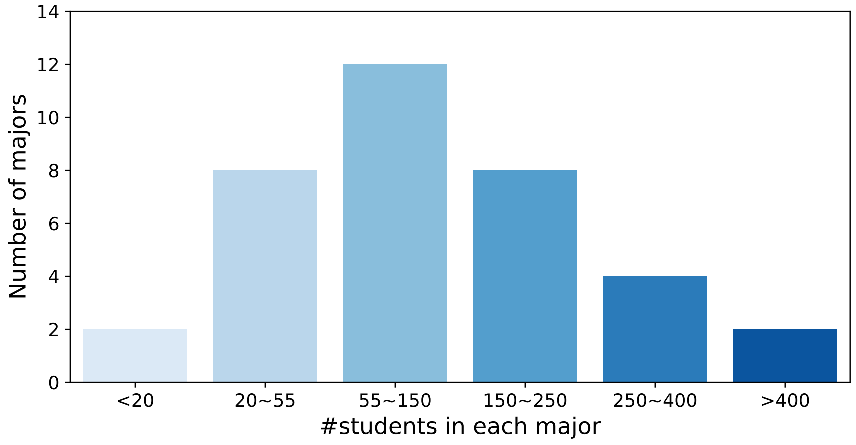

Supplementary Figure 4. Student compositions in each major or dorm room. Distribution of majors by the number of students per major. Most majors have 55 to 150 students. One major has fifteen students at the minimum and as many as over 400 students in the sample.

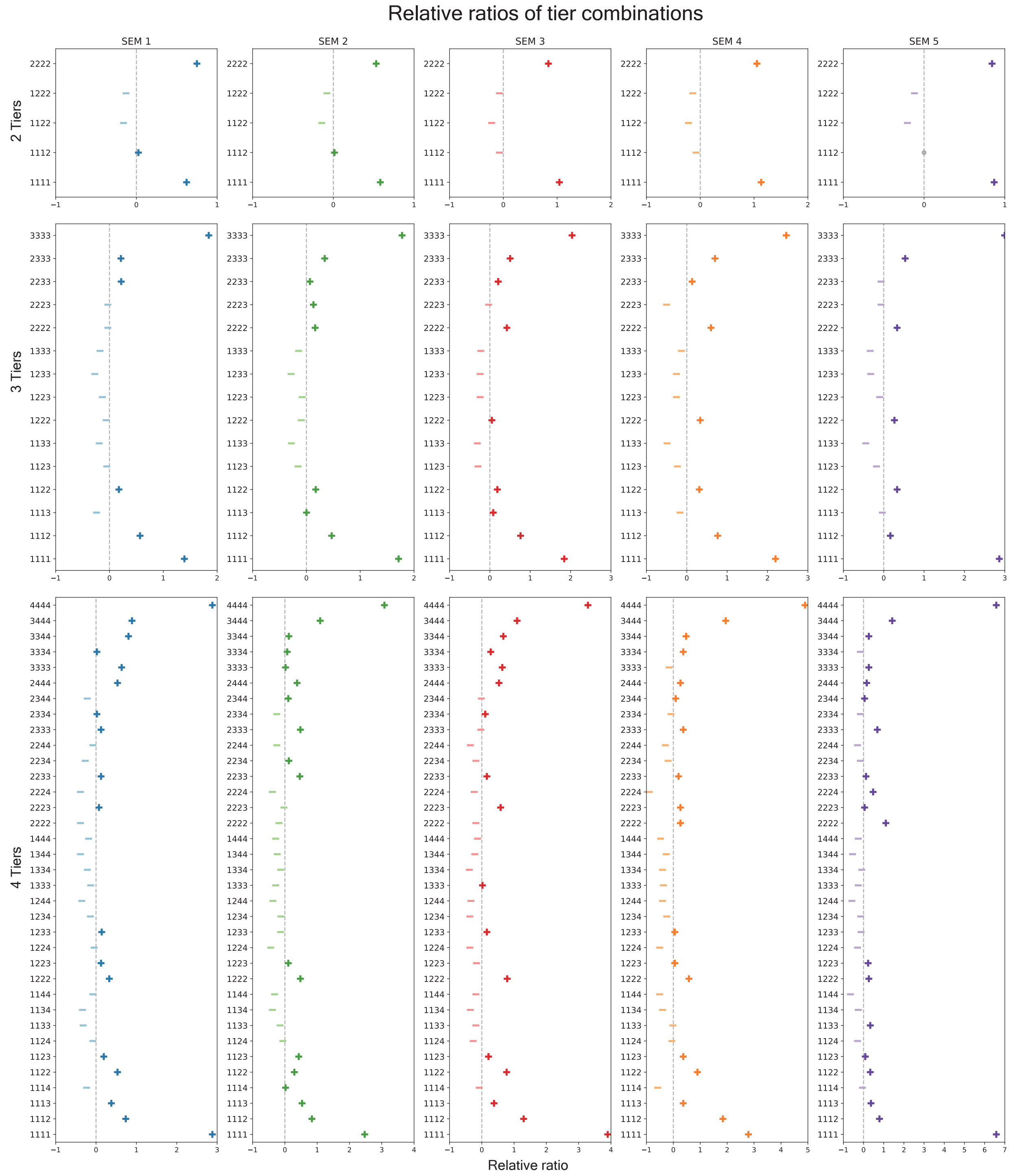

Supplementary Figure 5. The relative ratio $\mathbb{E}$ of each tier combination in each semester. Each row shows the $\mathbb{E}$ of each combination under the 2-tier, 3-tier, and 4-tier classification of GPA, respectively. Each column corresponds to one semester, from semester 1 to semester 5. The vertical axis shows all unique combinations in ascending order of tier numbers under a given tier of classification, and the horizontal axis shows $\mathbb{E}$ which compares the actual and theoretical probability of the same combination. The vertical dashed line marks $E = 0$, where the actual and theoretical probabilities are equal. Positive and negative E are marked by '+' and '-', respectively.

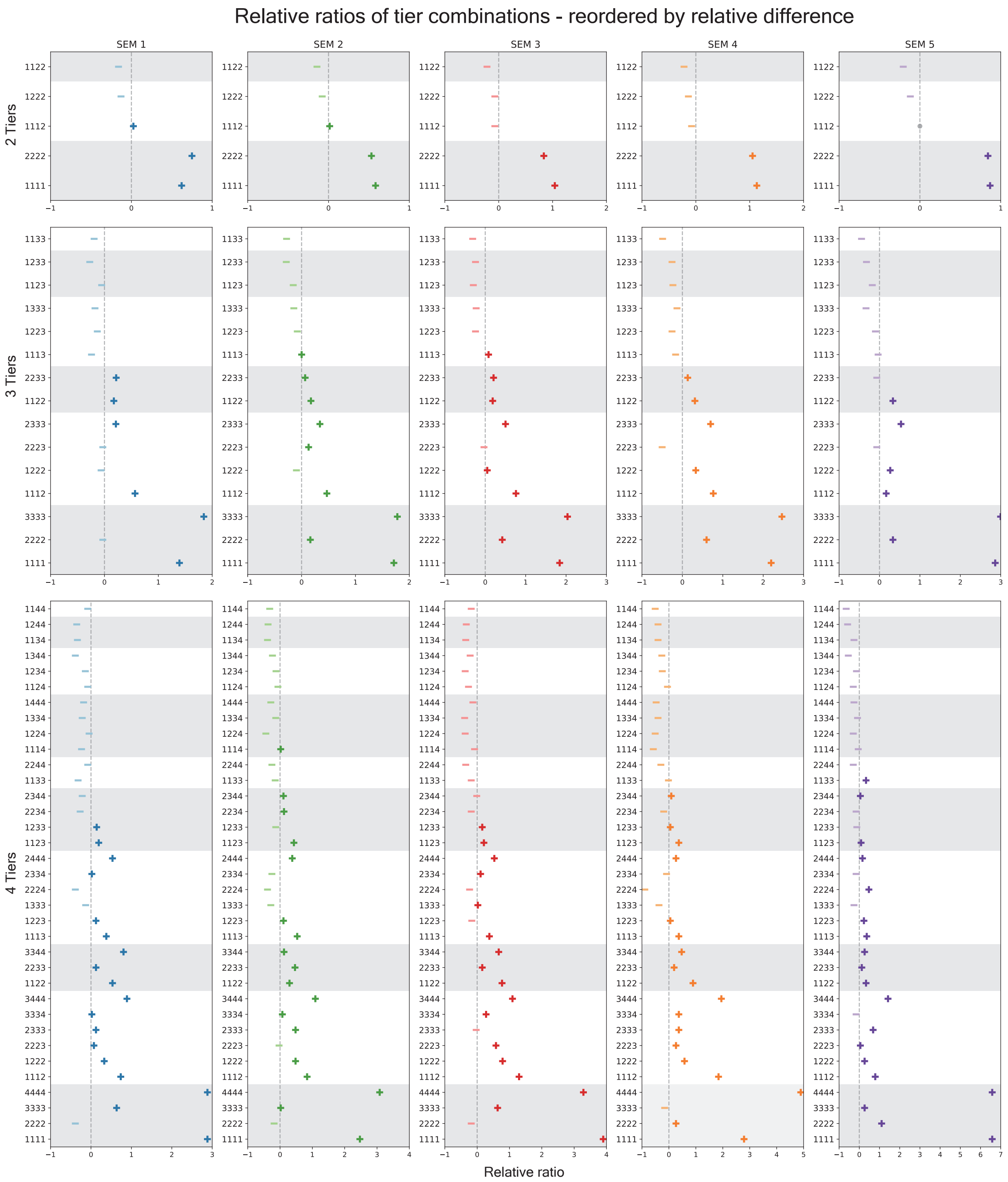

Supplementary Figure 6. Rearranged tier combinations according to their relative difference. The vertical axis shows combinations in ascending order of the relative difference *D*, which measures the average pairwise difference among tier numbers. The staggered gray shade marks a group of combinations with the same *D*. The horizontal axis shows $\mathbb{E}$ which compares the actual probability with the theoretical probability of the same combination. Other captions are the same as those for Supplementary Figure 5.

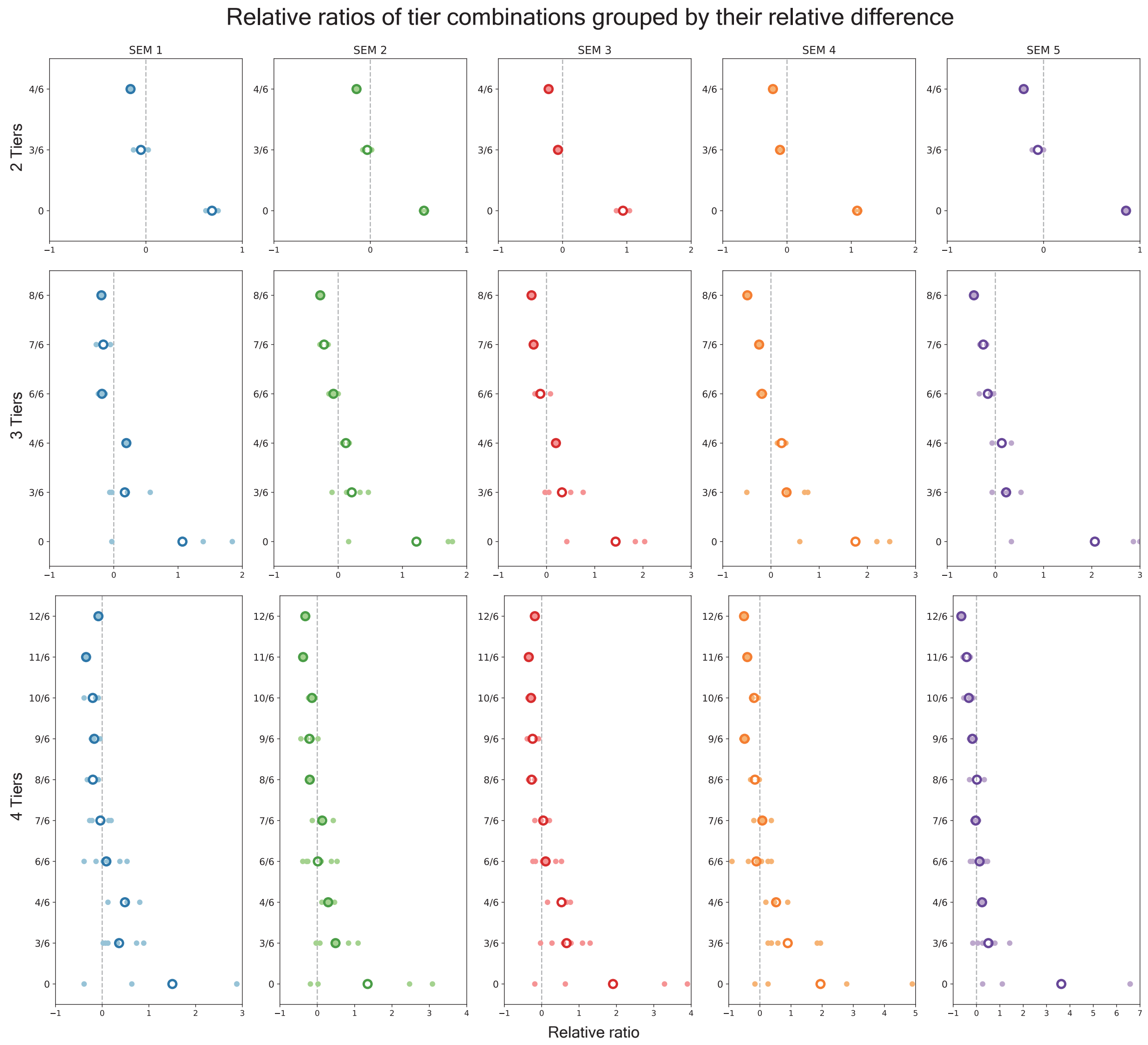

Supplementary Figure 7. The negative association between the relative ratio $\mathbb{E}$ and the relative difference *D*. The horizontal axis shows the relative ratio $\mathbb{E}$, and the vertical axis shows the relative difference D. Combinations with the same relative difference D are grouped and their relative ratios $\mathbb{E}$ are averaged. The hollow circle shows the mean $\mathbb{E}$ for each group with the same D, and data points show $\mathbb{E}$ for each combination. Other captions are the same as those for Supplementary Figure 5.

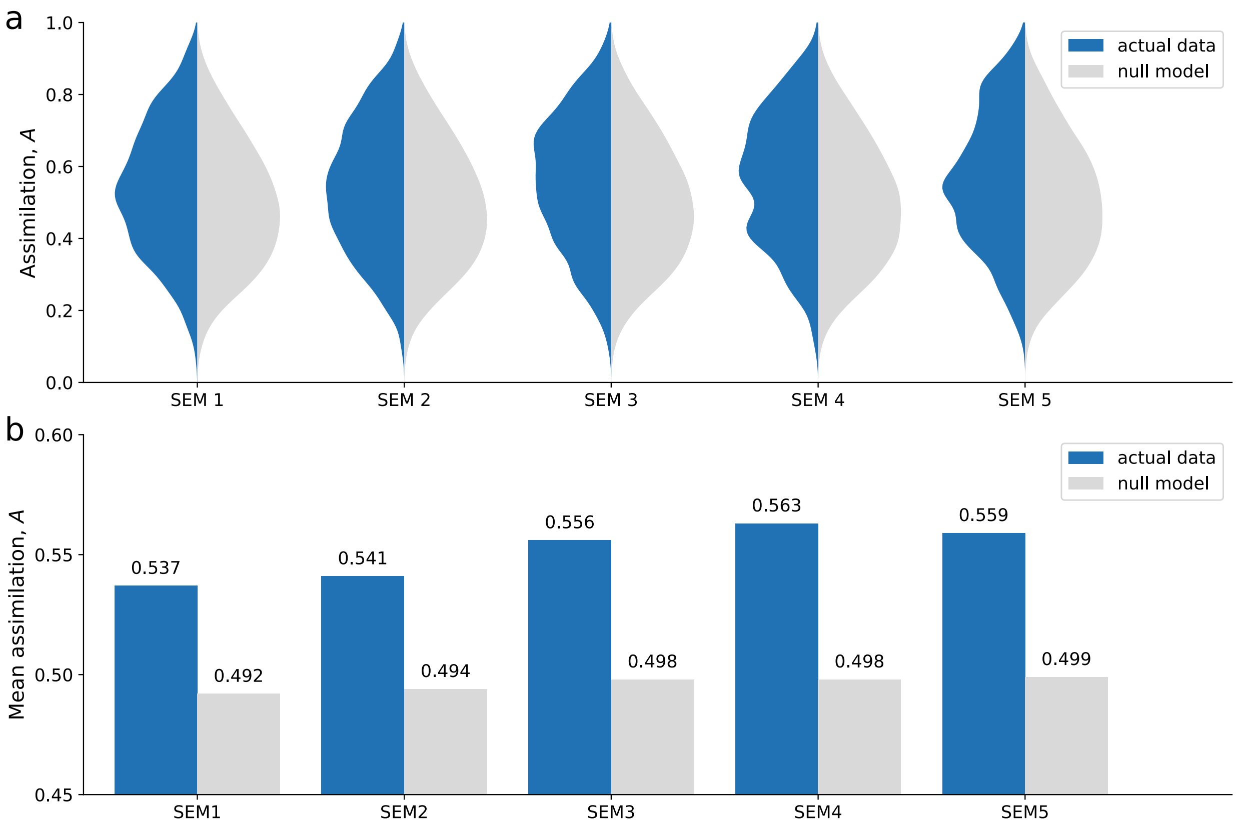

Supplementary Figure 8. The assimilation of roommate academic performance in each semester. (a) The density distribution *p(A)* of assimilation for all dorm rooms. In the distribution plot, the left half (in blue) shows the actual assimilation and the right half (in gray) shows the null-model assimilation. (b) The comparison between the mean actual assimilation and the mean null-model assimilation from semester 1 to semester 5. The mean value is labeled on each bar.

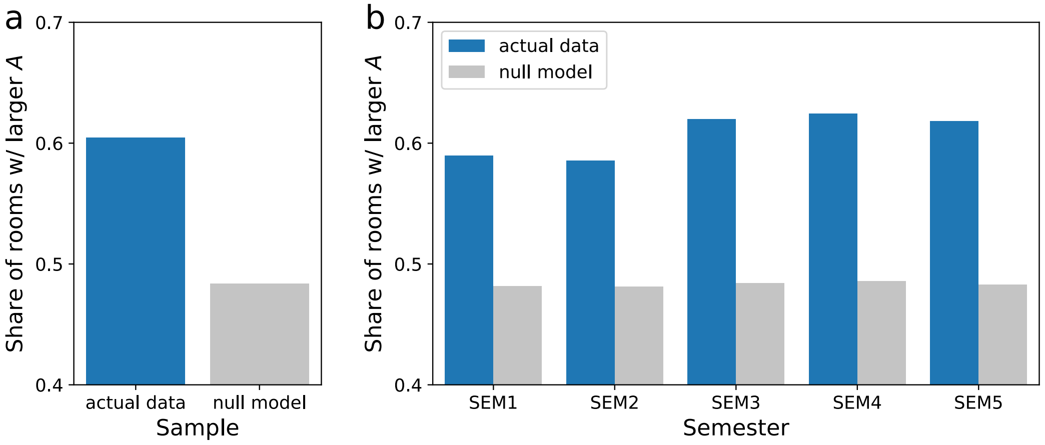

Supplementary Figure 9. The assimilation of roommate academic performance and its temporal trend. (a) The share of dorm rooms with larger-than-null-model assimilation according to the 95% confidence interval. (b) The results for each of the five semesters.

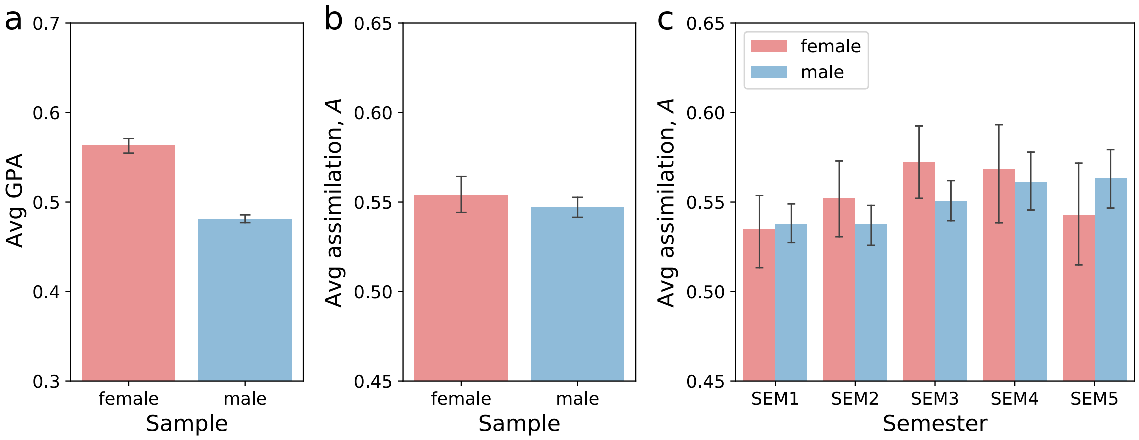

Supplementary Figure 10. The roommate peer effects for female and male students. (a) The average GPA for all five semesters. Error bars represent standard errors clustered for students. (b) The average assimilation for all five semesters. (c) The average assimilation for each of the five semesters. Error bars represent standard errors clustered for dorm rooms.

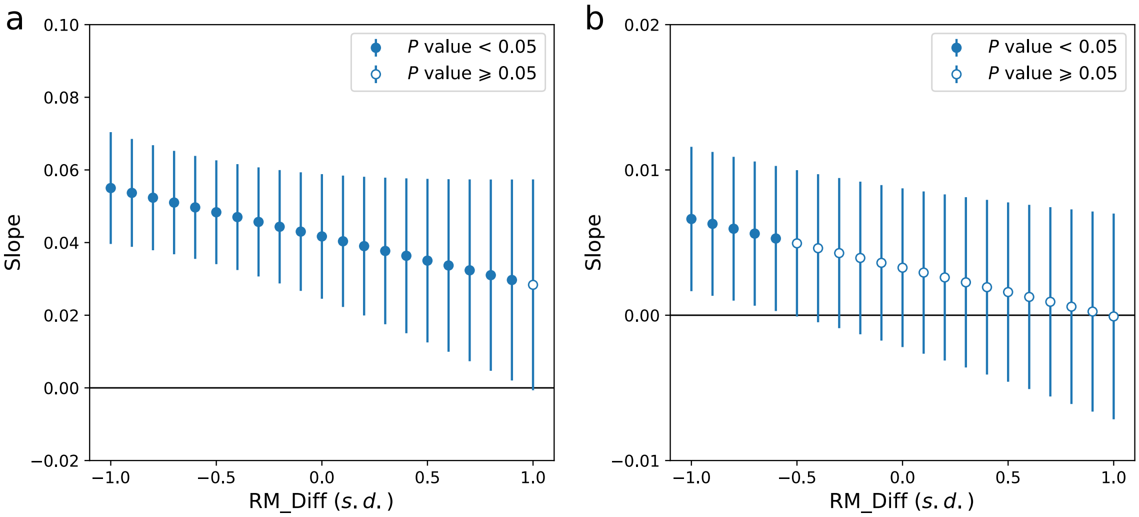

Supplementary Figure 11. Results of the simple slope tests on moderation effects. (a) The slope for the relationship between a student's post-GPA and their roommate average prior GPA. The relationship is moderated by the differences in roommate prior GPAs (RM_Diff), and the slope is significantly above 0 (P value < 0.1). (b) The slope for the relationship between a student's post-GPA and their in-dorm ordinal rank (OR_InDorm). The relationship is moderated by RM_Diff, but only when RM_Diff < −0.5 *s.d.*, the slope is significantly above 0 (P value < 0.05). In both panels, the x-axis shows the differences ranging from −1 *s.d.* to 1 *s.d.* of RM_Diff, and the y-axis shows the slope of the corresponding relationship. Solid circles show a P value < 0.05, and hollow circles show a P value ≥ 0.05. Error bars represent the 95% confidence intervals.

a

Variables	Dependent variable: GPA_Post				
	(1)	(2)	(3)	(4)	(5)
RM_Avg	0.046***	0.004***		0.004***	0.003***
	(0.001)	(0.001)		(0.001)	(0.001)
RM_Diff			0.001	0.001	0.001
			(0.001)	(0.001)	(0.001)
RM_Avg × RM_Diff					−0.011*
					(0.006)
GPA_Prior		0.809***	0.809***	0.809***	0.809***
		(0.000)	(0.000)	(0.000)	(0.000)
D (gender)		0.042***	0.043***	0.043***	0.043***
		(0.000)	(0.000)	(0.000)	(0.000)
D (semester)	No	Yes	Yes	Yes	Yes
Major FE	No	Yes	Yes	Yes	Yes
Cohort FE	No	Yes	Yes	Yes	Yes
Observations	1,568,000	1,568,000	1,568,000	1,568,000	1,568,000
Adjust R^2	0.001	0.667	0.667	0.667	0.667
RMSE	0.292	0.169	0.169	0.169	0.169

Robust standard errors in parentheses. *** p<0.01, ** p<0.05, * p<0.1

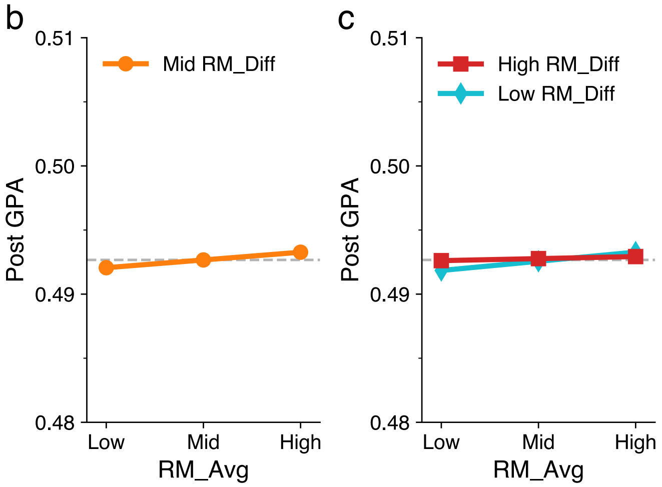

Supplementary Figure 12. Results of the falsification test for the effects of roommate average prior performance. The OLS regression is applied to the roommate null model that consists of 100 independent implementations. (a) Full regression results. (b) The margin plot for the linear relationship between GPA_Post and RM_Avg. (c) The margin plot for the moderating effects of RM_Diff on the relationship between GPA_Post and RM_Avg. The "Low" and "High" on the horizontal axis represent 1 standard deviation (*s.d.*) below and above the mean ("Mid") of RM_Avg, respectively. The "Low" and "High" in the legend represent 1 *s.d.* below and above the mean ("Mid") of RM_Diff, respectively. The horizontal dashed line marks the regression constant.

a

Variables	Dependent variable: GPA_Post				
	(1)	(2)	(3)	(4)	(5)
OR_InDorm	0.001***	0.001**	0.001**	0.001**	−0.000
	(0.000)	(0.000)	(0.000)	(0.000)	(0.000)
RM_Avg		0.002	0.002	0.002	0.002**
		(0.001)	(0.001)	(0.001)	(0.001)
RM_Diff		0.001	0.002*	0.001	0.001
		(0.001)	(0.001)	(0.001)	(0.001)
OR_InDorm × GPA_Prior			−0.003***		
			(0.000)		
OR_InDorm × RM_Avg				0.001**	
				(0.001)	
OR_InDorm × RM_Avg					−0.007***
					(0.001)
GPA_Prior	0.811***	0.811***	0.811***	0.811***	0.809***
	(0.001)	(0.001)	(0.001)	(0.001)	(0.001)
D (gender & semester)	Yes	Yes	Yes	Yes	Yes
Major & cohort FE	Yes	Yes	Yes	Yes	Yes
Observations	1,568,000	1,568,000	1,568,000	1,568,000	1,568,000
Adjust R^2	0.667	0.667	0.667	0.667	0.667
RMSE	0.169	0.169	0.169	0.169	0.169

Robust standard errors in parentheses. *** p<0.01, ** p<0.05, * p<0.1

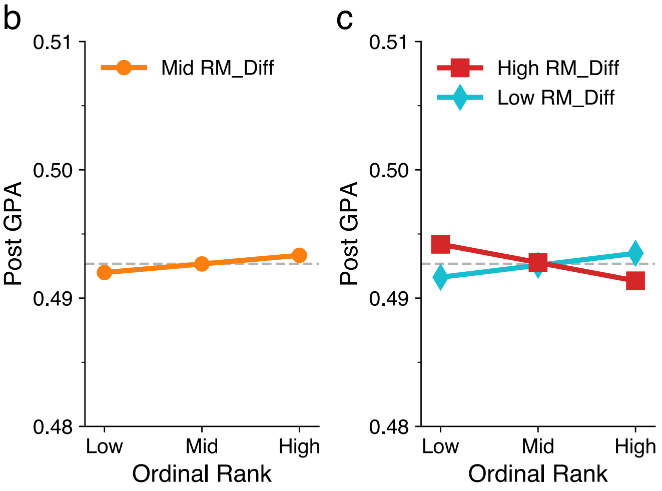

Supplementary Figure 13. Results of the falsification test for the effects of within-dorm ordinal rank. The OLS regression is applied to the roommate null model that consists of 100 independent implementations. (a) Full regression results. (b) The margin plot for the linear relationship between GPA_Post and OR_InDorm, where a larger value means a relatively low GPA among roommates. (c) The margin plot for the moderating effect of RM_Diff on the relationship between GPA_Post and OR_InDorm. The "Low" and "High" on the horizontal axis represent 1 standard deviation (*s.d.*) below and above the mean ("Mid") of OR_InDorm, respectively. The "Low" and "High" in the legend represent 1 *s.d.* below and above the mean ("Mid") of RM_Diff, respectively. Horizontal dashed lines mark regression constants.

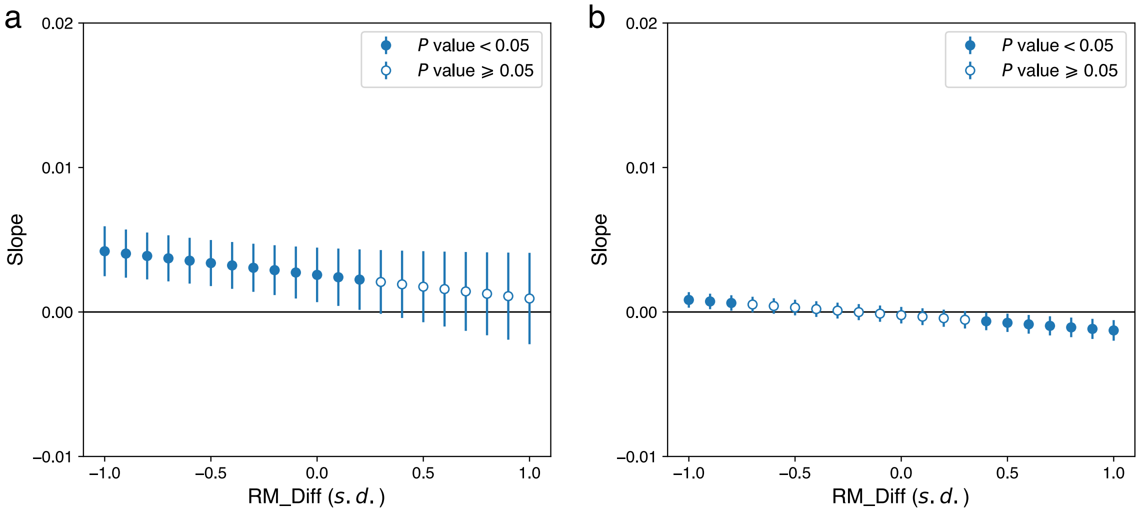

Supplementary Figure 14. Results of the simple slope tests on moderation effects. (a) The slope for the relationship between a student's post-GPA and their roommate average prior GPA. The relationship is moderated by the differences in roommate prior GPAs (RM_Diff), and the slope is significantly above 0 only when RM_Diff < 0.3 *s.d.* (P value < 0.05). (b) The slope for the relationship between a student's post-GPA and their in-dorm ordinal rank (OR_InDorm). The relationship is moderated by RM_Diff. The slope is significantly above 0 when RM_Diff < −0.7 *s.d.* and significantly below 0 when RM_Diff > 0.3 *s.d.* (P value < 0.05). In both panels, the x-axis shows the differences ranging from −1 *s.d.* to 1 *s.d.* of RM_Diff, and the y-axis shows the slope of the corresponding relationship. Solid circles show a P value < 0.05, and hollow circles show a P value ≥ 0.05. Error bars represent the 95% confidence intervals.

Supplementary Tables

Supplementary Table 1. The theoretical probability of each tier combination.

Tier	1111	1112	1113	1114	1122	1123	1124	1133	1134	1144	1222	1223
2	1/16	1/4	–	–	3/8	–	–	–	–	–	1/4	–
3	1/81	4/81	4/81	–	2/27	4/27	–	2/27	–	–	4/81	4/27
4	1/256	1/64	1/64	1/64	3/128	3/64	4/64	3/128	3/64	3/128	1/64	3/64
**Tier**	**1224**	**1233**	**1234**	**1244**	**1333**	**1334**	**1344**	**1444**	**2222**	**2223**	**2224**	**2233**
2	–	–	–	–	–	–	–	–	1/16	–	–	–
3	–	4/27	–	–	4/81	–	–	–	1/81	4/81	–	2/27
4	3/64	3/64	3/32	3/64	1/64	3/64	3/64	1/64	1/256	1/64	1/64	3/128
**Tier**	**2234**	**2244**	**2333**	**2334**	**2344**	**2444**	**3333**	**3334**	**3344**	**3444**	**4444**	
2	–	–	–	–	–	–	–	–	–	–	–	
3	–	–	4/81	–	–	–	1/81	–	–	–	–	
4	3/64	3/128	1/64	3/64	3/64	1/64	1/256	1/64	3/128	1/64	1/256	

Notes: The total number of combinations are 5, 15, and 35 when applying 2-tier, 3-tier, and 4-tier classifications, respectively. A combination has no particular tier order, so the one in ascending tier order is used to delegate all identical others. Each title row shows combinations, and the value in the table is the theoretical probability of each combination, respectively. The symbol "–" means that there is no such combination under the corresponding tier classification.

Supplementary Table 2. The significance of each tier combination in the actual data.

Tier	1111	1112	1113	1114	1122	1123	1124	1133	1134	1144	1222	1223
2	***	(***)	–	–	(***)	–	–	–	–	–	(**)	–
3	***	***	(**)	–	**	(***)	–	(***)	–	–	n.s.	(***)
4	***	***	***	(*)	***	n.s.	(***)	(**)	(***)	(***)	*	n.s.
**Tier**	**1224**	**1233**	**1234**	**1244**	**1333**	**1334**	**1344**	**1444**	**2222**	**2223**	**2224**	**2233**
2	–	–	–	–	–	–	–	–	***	–	–	–
3	–	(***)	–	–	n.s.	–	–	–	***	n.s.	–	***
4	(***)	(**)	(***)	(***)	(*)	(***)	(**)	n.s.	*	***	n.s.	***
**Tier**	**2234**	**2244**	**2333**	**2334**	**2344**	**2444**	**3333**	**3334**	**3344**	**3444**	**4444**	
2	–	–	–	–	–	–	–	–	–	–	–	
3	–	–	***	–	–	–	***	–	–	–	–	
4	n.s.	(*)	**	n.s.	**	***	n.s.	***	***	***	***	

Notes: A statistical test is performed using the roommate null model to examine the significance of each combination's relative difference $\mathbb{E}$ under the 2-tier, 3-tier, and 4-tier classifications, respectively (see Section 3.2). The null model consists of 1000 independent implementations, and the statistical test is one-sided. Each title row shows combinations. The value in the table marks the significance of the combination's $\mathbb{E}$, where *, **, and *** represent significantly positive $\mathbb{E}$ with a P value < 0.1, 0.05, and 0.01, respectively. The symbol "(·)" means significantly negative $\mathbb{E}$ at the corresponding level, "n.s." means not significantly different from 0 (i.e., P value > 0.1), and "–" means that there is no such combination under the corresponding tier classification.

Supplementary Table 3. Summary statistics of variables and their correlations.

No.	Variable	Symbol	Mean	Std. Dev.	Pearson's correlation coefficient							
					1	2	3	4	5	6	7	8
1	GPA_Post	G^{s+1}	0.500	0.292								
2	GPA_Prior	G^{s}	0.500	0.292	0.815							
3	RM_Avg	RA^{s}	0.500	0.195	0.244	0.251						
4	RM_Diff	RD^{s}	0.303	0.150	0.020	0.020	0.024					
5	OR_InDorm	OR^{s}	2.500	1.118	−0.539	−0.694	0.347	−0.000				
6	D (gender)	D^{Ge}	0.226	0.418	0.132	0.108	0.162	−0.009	0.000			
7	D (major)	D^{Ma}	10.750	8.562	0.000	0.000	−0.007	0.045	−0.005	0.088		
8	D (cohort)	D^{Co}	1.345	0.475	0.000	−0.000	0.000	0.018	0.000	0.019	−0.013	
9	D (semester)	D^{Se}	2.155	1.064	0.000	−0.000	−0.000	−0.043	−0.000	−0.009	0.006	−0.447

Notes: GPA_Post and GPA_Prior are the student's GPA in semester s+1 and s, respectively. The semester index s ranges from 1 to 4. RM_Avg is the average roommate prior GPA. RM_Diff is the differences in roommate prior GPAs. OR_InDorm is the student's in-dorm ordinal rank with 1 being the highest and 4 being the lowest in ranking (i.e., from the highest to the lowest academic performance). The gender dummy D (gender) is coded as 1 and 0 for females and males, respectively. The major dummy D (major) is coded from 1 to 36 from the largest to the smallest major according to the number of students. The cohort dummy D (cohort) is coded as 1 and 2 for the 2011 and 2012 cohort, respectively. The semester dummy D (semester) is coded from 1 to 4 from the first to the fourth semester. Data from all five semesters are pooled together.
