# Supplementary material for: Heterogeneous peer effects of college roommates on academic performance
Source: Nat Commun. 2024 Jun 6;15:4785. doi: 10.1038/s41467-024-49228-7 (PMC11156860; doi:10.1038/s41467-024-49228-7)
Supplement: Supplementary file 3 — Reporting summary [file 41467_2024_49228_MOESM3_ESM.pdf]

nature portfolio

Corresponding author(s): Tao Zhou; Jian Gao

Last updated by author(s): May 15, 2024

Reporting Summary

Nature Portfolio wishes to improve the reproducibility of the work that we publish. This form provides structure for consistency and transparency in reporting. For further information on Nature Portfolio policies, see our Editorial Policies and the Editorial Policy Checklist.

Statistics

For all statistical analyses, confirm that the following items are present in the figure legend, table legend, main text, or Methods section.

n/a	Confirmed	
☐	☒	The exact sample size (*n*) for each experimental group/condition, given as a discrete number and unit of measurement
☐	☒	A statement on whether measurements were taken from distinct samples or whether the same sample was measured repeatedly
☐	☒	The statistical test(s) used AND whether they are one- or two-sided<br>*Only common tests should be described solely by name; describe more complex techniques in the Methods section.*
☐	☒	A description of all covariates tested
☐	☒	A description of any assumptions or corrections, such as tests of normality and adjustment for multiple comparisons
☐	☒	A full description of the statistical parameters including central tendency (e.g. means) or other basic estimates (e.g. regression coefficient) AND variation (e.g. standard deviation) or associated estimates of uncertainty (e.g. confidence intervals)
☐	☒	For null hypothesis testing, the test statistic (e.g. *F*, *t*, *r*) with confidence intervals, effect sizes, degrees of freedom and *P* value noted<br>*Give P values as exact values whenever suitable.*
☒	☐	For Bayesian analysis, information on the choice of priors and Markov chain Monte Carlo settings
☐	☒	For hierarchical and complex designs, identification of the appropriate level for tests and full reporting of outcomes
☐	☒	Estimates of effect sizes (e.g. Cohen's *d*, Pearson's *r*), indicating how they were calculated

Our web collection on statistics for biologists contains articles on many of the points above.

Software and code

Policy information about availability of computer code

Data collection	The undergraduate dataset was obtained under a data use agreement, and no code was used to collect the raw data. Details about the data are provided in the Supplementary Information file.
Data analysis	Data was analyzed with customized code in Python 3 using standard software packages within these programs including matplotlib, seaborn, NumPy, statsmodels, and others. All code necessary to replicate the statistical analyses and main figures in this paper has been deposited in the freely available data repository at Figshare (https://www.doi.org/10.6084/m9.figshare.25286017).

For manuscripts utilizing custom algorithms or software that are central to the research but not yet described in published literature, software must be made available to editors and reviewers. We strongly encourage code deposition in a community repository (e.g. GitHub). See the Nature Portfolio guidelines for submitting code & software for further information.

Data

Policy information about availability of data

All manuscripts must include a data availability statement. This statement should provide the following information, where applicable:

- Accession codes, unique identifiers, or web links for publicly available datasets
- A description of any restrictions on data availability
- For clinical datasets or third party data, please ensure that the statement adheres to our policy

The raw data of anonymized student accommodation and academic performance are protected by a data use agreement. Those who are interested in the raw data

may contact the corresponding authors for access after obtaining Institutional Review Board (IRB) approval. All data necessary to replicate the statistical analyses and main figures in this paper are available in Supplementary Information and have been deposited in the freely available data repository at Figshare (https://www.doi.org/10.6084/m9.figshare.25286017).

Research involving human participants, their data, or biological material

Policy information about studies with human participants or human data. See also policy information about sex, gender (identity/presentation), and sexual orientation and race, ethnicity and racism.

Reporting on sex and gender	The analyzed sample covers both female and male students.
Reporting on race, ethnicity, or other socially relevant groupings	This study doesn't involve data on race, ethnicity, or other socially relevant groupings.
Population characteristics	This study covers undergraduates from cohorts 2011 and 2012 in a world-class research university in China during 2011-2014.
Recruitment	There was no recruitment in this study.
Ethics oversight	This study was approved by the Institutional Review Board (IRB) at the University of Electronic Science and Technology of China (IRB No. 1061420210802005). This information has been included in the Methods section of the paper.

Note that full information on the approval of the study protocol must also be provided in the manuscript.

Field-specific reporting

Please select the one below that is the best fit for your research. If you are not sure, read the appropriate sections before making your selection.

☐ Life sciences ☒ Behavioural & social sciences ☐ Ecological, evolutionary & environmental sciences

For a reference copy of the document with all sections, see nature.com/documents/nr-reporting-summary-flat.pdf

Behavioural & social sciences study design

All studies must disclose on these points even when the disclosure is negative.

Study description	This study investigates peer effects among college roommates with random assignments on academic performance based on the longitudinal data covering two cohorts of undergraduates for five semesters. The tier combinations of roommates after coarsening their GPA rankings into tiers were examined and showed that the probabilities of tier combinations significantly deviate from their theoretical values. An assimilation metric based on roommates GPA rankings was proposed and corresponding null models were performed to demonstrate the presence and trend of roommate peer effects. An ordinary least squares (OLS) regression model was employed to study peer effects along the dimension of peer heterogeneity.
Research sample	This study covers undergraduates from a world-class research university in mainland China during 2011-2014. Students were randomly assigned to identical 4-person dorm rooms before registration for the first semester of Freshman year. The Housing Office of the university neither offers students the option to choose roommates nor has access to their academic and socioeconomic background data. Once assigned to a dorm room, four students usually live together for the entire four years of undergraduate programs until their graduation in principle. The final data set covers 5,272 undergraduates in 4-person dorm rooms from the 2011 cohort and the 2012 cohort as well as their academic performance.
Sampling strategy	The sample is based on digital records of undergraduates' on-campus accommodation information and academic performance in an A-level university in Mainland China. We filtered undergraduates who came from the cohorts 2011 and 2012 and lived in 4-person dorm rooms without room changing. For comparison across different majors and cohorts, we transformed each student's GPA into a percentile R among students of the same major and cohort for each semester.
Data collection	The raw undergraduate data was originally collected from an educational-oriented big data integration solution provider. Our dataset was obtained under a data use agreement, and the raw data was anonymized and de-identified.
Timing	The raw undergraduates data was originally collected in 2015. The obtained anonymous and de-identified dataset was originally collected in 2017.
Data exclusions	The study only considers undergraduates who lived in 4-person dorm rooms without room changing during the considered period. The undergraduates whose academic performance was not available were excluded at the time of data analysis.
Non-participation	N/A
Randomization	There was no randomization, given the observational nature of this study.

Reporting for specific materials, systems and methods

We require information from authors about some types of materials, experimental systems and methods used in many studies. Here, indicate whether each material, system or method listed is relevant to your study. If you are not sure if a list item applies to your research, read the appropriate section before selecting a response.

Materials & experimental systems

n/a	Involved in the study
☒	☐ Antibodies
☒	☐ Eukaryotic cell lines
☒	☐ Palaeontology and archaeology
☒	☐ Animals and other organisms
☒	☐ Clinical data
☒	☐ Dual use research of concern
☒	☐ Plants

Methods

n/a	Involved in the study
☒	☐ ChIP-seq
☒	☐ Flow cytometry
☒	☐ MRI-based neuroimaging
